# Influence of cell culture-derived media and environmental contaminants on the effect of feline calicivirus inactivation agents

**DOI:** 10.1038/s41598-025-05311-7

**Published:** 2025-07-01

**Authors:** Rei Saito, Shiori Katta, Takeshi Takizawa, Junichi Sugiyama, Yasushi Kakizawa, Shigeru Morikawa

**Affiliations:** 1 Advanced Analytical Science Research Laboratories, Lion Corporation, 7-2-1 Hirai, Edogawa-ku, Tokyo, 132-0035 Japan; 2 Faculty of Veterinary Medicine, Okayama University of Science, 1-3 Ikoinooka, Imabari City, Ehime Japan; 3 National Institute of Infectious Diseases, 1-23-1 Toyama, Shinjyuku-ku, Tokyo, 162-8640 Japan

**Keywords:** Non-enveloped virus, Sodium dodecyl sulfate, Didecyl dimethylammonium chloride, Surfactant, Ethanol, Sodium hypochlorite, Inactivation effect, Virus suspension, Environmental contaminants, Inactivation mechanism, Chemical biology, Microbiology, Chemistry

## Abstract

Several studies on viral inactivation agents frequently overlook the influence of virus suspension conditions, which often leads to inconsistent conclusions. We investigated the impact of cell culture-derived media and environmental contaminants on the efficacy of viral inactivation agents against feline calicivirus (FCV). The FCV in Eagle’s minimum essential medium (EMEM) with cell-derived metabolites, was replaced with distilled water (DW) with using a column-based simple dispersant replacement method. Sodium dodecyl sulfate (SDS) or sodium hypochlorite (NaClO) were less effective against FCV in EMEM than DW, while didecyl dimethylammonium chloride (DDAC) was more effective. The inactivation effect of 50% v/v ethanol was stronger against FCV in EMEM than DW, whereas that of 70% v/v ethanol was more effective in DW. Analysis of the effect of EMEM components revealed that inorganic salts and basic amino acids (BAA) reduced SDS inactivation efficacy but enhanced DDAC’s. Similarly, inorganic salts reduced the effect of 70% v/v ethanol, while amino acids, especially BAA reduced that of NaClO’s. Since each inactivating agent affects FCV differently, the influence of each EMEM component is assumed to differ. Moreover, environmental contaminants, such as bovine serum and saliva, reduced the overall effectiveness of the agents owing to their inorganic substances and proteins.

**Supplementary Information:**

The online version contains supplementary material available at 10.1038/s41598-025-05311-7.

## Introduction

Information regarding the efficacy of disinfectants and their active ingredients against viruses is critical, particularly during a pandemic^1^. Various surfactants, ethanol and sodium hypochlorite (NaClO) have been identified as effective agents for the effective inactivation of enveloped viruses, such as SARS-CoV-2 and influenza viruses, which cause seasonal epidemics^2–4^. However, there are cases wherein the efficacy of inactivation vary among reports. For instance, Guo et al.^5^ reported that 16% ethanol reduced the abundance of SARS-CoV-2 by a reduction of infectivity titer in log_10_ (Δlog) = 1.75, while Nomura et al.^6^ reported that 18% ethanol had no antiviral effect. Such variations in reported effectiveness can occur even for the same disinfectant. Additionally, viruses can be classified as enveloped and non-enveloped viruses, with non-enveloped viruses being more resistant to inactivation due to their outer protein shell, known as a capsid^7^. Therefore, a more accurate assessment of efficacy is essential. However, as with enveloped viruses, evaluation results can vary even for the same agents. For example, Sanekata et al. reported that 100 ppm NaClO could inactivate more than 99.99% of feline calicivirus (FCV), a surrogate virus for noroviruses^8^. However, Erwin et al. reported that 300 ppm NaClO could only inactivate approximately 10% of FCV^9^. Aken et al. used machine learning to determine the reason for the difference in virus inactivation efficacy using the same disinfectant^10^. They indicated that this difference was due to the presence or absence of organic substances in the viral suspension, regardless of the virus type. When evaluating disinfectant efficacy, the American Society for Testing and Materials (ASTM) and European Committee for Standardization (CEN) recommend the use of bovine serum albumin (BSA) and other substances as environmental contaminants to simulate actual conditions, because proteins can interfere with their efficacy^11–13^. Therefore, the effects of disinfectants presumably vary according to the test method used as a reference. Furthermore, since viruses generally infect and propagate in the cells, the virus suspension contains cell culture-derived metabolites, culture media, and other components. The cell type and culture media conditions vary depending on the virus type, and the components in the virus suspension also differ depending on the subsequent virus purification method. Further, pathogens are often contaminated by blood or saliva in a real environment.

In this study, we aimed to clarify the influence of medium-derived dissolved components and other environmental contaminants in viral suspensions on the effectiveness of virus-inactivating agents used including general-purpose products such as disinfectants and surface cleaners, using non-enveloped FCV. Specifically, we removed dissolved components such as culture media and serum, from FCV and observed that the effect of the agents was highly dependent on the viral suspension conditions.

## Results

### The influence of eagle’s minimum essential medium (EMEM) on the effect of virus inactivation agents

The FCV inactivation effects of various agents investigated in this study were evaluated using FCV suspended in EMEM, a growth medium, and FCV in which the dispersion medium was replaced with distilled water (DW) using a PD-10 column simply (Table 1). The results showed that 0.5% w/v sodium dodecyl sulfate (SDS) did not exhibit an inactivation effect in EMEM but exhibited a strong effect in DW (Δlog ≥ 4.03). Didecyl dimethylammonium chloride (DDAC) displayed a strong inactivation effect at a concentration of 0.05% w/v in EMEM (Δlog ≥ 3.08). However, in DW, its inactivation effect decreased to approximately Δlog = 2. Similarly, 50% v/v ethanol in EMEM exhibited an inactivation effect of Δlog = 2.55, while its effectiveness remained consistent at a concentration of 70% v/v. In contrast, ethanol showed low efficacy in DW at a concentration of 50% v/v (Δlog = 0.99), but its effect significantly increased at a concentration of 70% v/v (Δlog = 4.00). For NaClO, an inactivation effect was observed at concentrations starting from 100 ppm in EMEM. However, in DW, it showed a strong inactivation effect (Δlog ≥ 4.03) at a much lower concentration of 10 ppm.

**Table 1:** Effect of the individual FCV inactivation agents.

Sample		Dispersion solvent of FCV	
Agent	Concentration	DW	EMEM
SDS	0.05% w/v	0.04 ± 0.09	0.20 ± 0.16
	0.10% w/v	0.15 ± 0.31	0.17 ± 0.31
	0.50% w/v	≥ 4.03*	0.20 ± 0.16
DDAC	0.01% w/v	1.23 ± 0.41	1.32 ± 0.19*
	0.05% w/v	2.04 ± 0.17*	≥ 3.08*
	0.10% w/v	2.40 ± 0.19*	≥ 3.08*
Ethanol	30% v/v	0.15 ± 0.02	0.14 ± 0.45
	50% v/v	0.99 ± 0.06*	2.55 ± 0.24*
	70% v/v	4.00 ± 0.05*	2.38 ± 0.24*
NaClO^a)^	10 ppm	≥ 4.03*	0.46 ± 0.37
	50 ppm	≥ 4.03*	1.45 ± 0.37
	100 ppm	≥ 4.03*	≥ 4.08*

The contact time was 10 min, and the inactivation effect (Δlog) was expressed as the mean ± SD (*n* = 3).

a) The values shown indicate the free chlorine concentrations in the samples. *Indicates statistically significant differences (*p* ≤ 0.05).

*DDAC* dodecyl dimethylammonium chloride, *EMEM* Eagle’s Minimum Essential Medium, *FCV* feline calicivirus, *SDS* sodium dodecyl sulfate.

### Influence of the components of EMEM on the effect of virus inactivation agents

We evaluated the influence of each component of EMEM on the effects of the virus inactivation agents against FCV (Tables 2 and 3)^14^. The inorganic salts in EMEM decreased the efficacy of SDS and ethanol while increasing the efficacy of DDAC; however, they did not impact the efficacy of NaClO. Basic amino acids (BAA) decreased the efficacy of SDS and NaClO, and increased that of DDAC. Neutral amino acids (NAA) group did not significantly influence the effects of SDS, DDAC, or ethanol; however, they decreased the effect of NaClO. Glucose did not influence the effects of any of the agents investigated.

**Table 2:** Major components of eagle’s minimum essential medium (EMEM).

Group	Ingredients	Concentration	
		mg/L	mmol/L
Inorganic salts	CaCl_2_	200.0	1.8
	KCl	400.0	0.8
	MgSO_4_	98.0	5.4
	NaCl	6800.0	26.2
	NaHCO_3_	2200.0	116.4
	NaH_2_PO_4_	121.7	1.0
BAA	L-Arginine·HCl	126.0	0.6
	L-Histidine·HCl·H_2_O	42.0	0.2
	L-Lysine HCl	72.5	0.4
NAA	L-Cysteine	23.8	2.0
	L-Glutamine	292.0	0.1
	L-Isoleucine	52.0	0.4
	L-Leucine	52.0	0.4
	L-Methionine	15.0	0.1
	L-Phenylalanine	32.0	0.2
	L-Threonine	48.0	0.4
	L-Tryptophan	10.0	0.0
	L-Tyrosine	36.0	0.2
	L-Valine	46.0	0.4
Others	D-Glucose	1000.0	5.55

**Table 3:** Additive effect of each EMEM component group on the FCV inactivation agents.

Sample	ΔLog				
	Dispersion solvent of FCV				
	DW	Inorganic salts	BAA	NAA	Glucose
0.5% w/v SDS	≥ 4.37*	0.35 ± 0.33	1.50 ± 0.10*	≥ 4.44*	≥ 4.12*
0.05% w/v DDAC	1.80 ± 0.17*	3.44 ± 0.05*	2.85 ± 0.05*	2.00 ± 0.15*	1.84 ± 0.15*
70% v/v ethanol	≥ 4.08*	3.15 ± 0.05*	≥ 3.84*	3.97 ± 0.05*	3.72 ± 0.10*
10 ppm NaClO^a)^	≥ 4.07*	≥ 4.47*	1.25 ± 0.22*	3.16 ± 0.16*	≥ 4.03*

The contact time was 10 min, and the inactivation effect (Δlog) was expressed as the mean ± SD (*n* = 3).

a) The values shown indicate the effective chlorine concentrations in the samples. *﻿Indicates statistically significant differences (*p* ≤ 0.05).

*DDAC* dodecyl dimethylammonium chloride, *EMEM* Eagle’s Minimum Essential Medium, *FCV* feline calicivirus, *SDS* sodium dodecyl sulfate.

### Change in the critical micelle concentration (CMC) of surfactants with or without EMEM

The adsorption behavior of surfactants at the interface changed significantly at concentrations before and after reaching the CMC, with their CMCs varying depending on the dissolved components in EMEM. Therefore, the CMC of each evaluated sample was measured using a surface tension meter (Table 4). The CMC of SDS in EMEM was 0.052% w/v but increased to 0.21% w/v in DW. The CMC of DDAC was 0.015% w/v in EMEM, but increased to 0.065% w/v in DW.

**Table 4:** Critical micelle concentration (CMC) of surfactants in each dispersant.

Surfactant	SDS		DDAC	
Dispersant	Water	EMEM	Water	EMEM
mM	7.45	1.81	1.80	0.41
% w/v	0.21	0.052	0.065	0.015

*DDAC* dodecyl dimethylammonium chloride, *EMEM* Eagle’s Minimum Essential Medium, *FCV* feline calicivirus, *SDS* sodium dodecyl sulfate.

### Free Chlorine in a NaClO solution is consumed by dissolved components in EMEM and environmental contaminants

The concentration of chlorine consumed in the evaluation solution was also measured, as microorganisms are inactivated via oxidation with the free chlorine in NaClO (Table 5). Chlorine was not consumed in the presence of glucose or inorganic salt groups, but was consumed in the presence of the other components.

**Table 5:** Chlorine concentration consumed by dissolved components in EMEM and environmental contaminants carried from the dispersants.

Mixture Components	EMEM	BAA	NAA	Glucose	Inorganic salts	0.03% BSA	5% FBS	Model saliva
Concentration of chlorine consumed (ppm)	78.0 ± 3.6*	7.7 ± 1.7*	15.0 ± 1.6*	0.7 ± 0.9	− 0.3 ± 1.2	6.3 ± 1.2*	363.3 ± 4.7^a),*^	110.7 ± 1.7*

The contact time was 10 min, and the results are expressed as the mean ± SD (*n* = 3).

a) Accurate measurements were not possible with 5% serum as an admixture, so this result was calculated by multiplying the measured value with 0.5% FBS by 10. *﻿Indicates statistically significant differences (*p* ≤ 0.05).

*BSA* bovine serum albumin, *EMEM* Eagle’s Minimum Essential Medium, *FBS* fetal bovine serum.

### Inactivation effect of agents in the presence of environmental contaminants

**Fig. 1:**
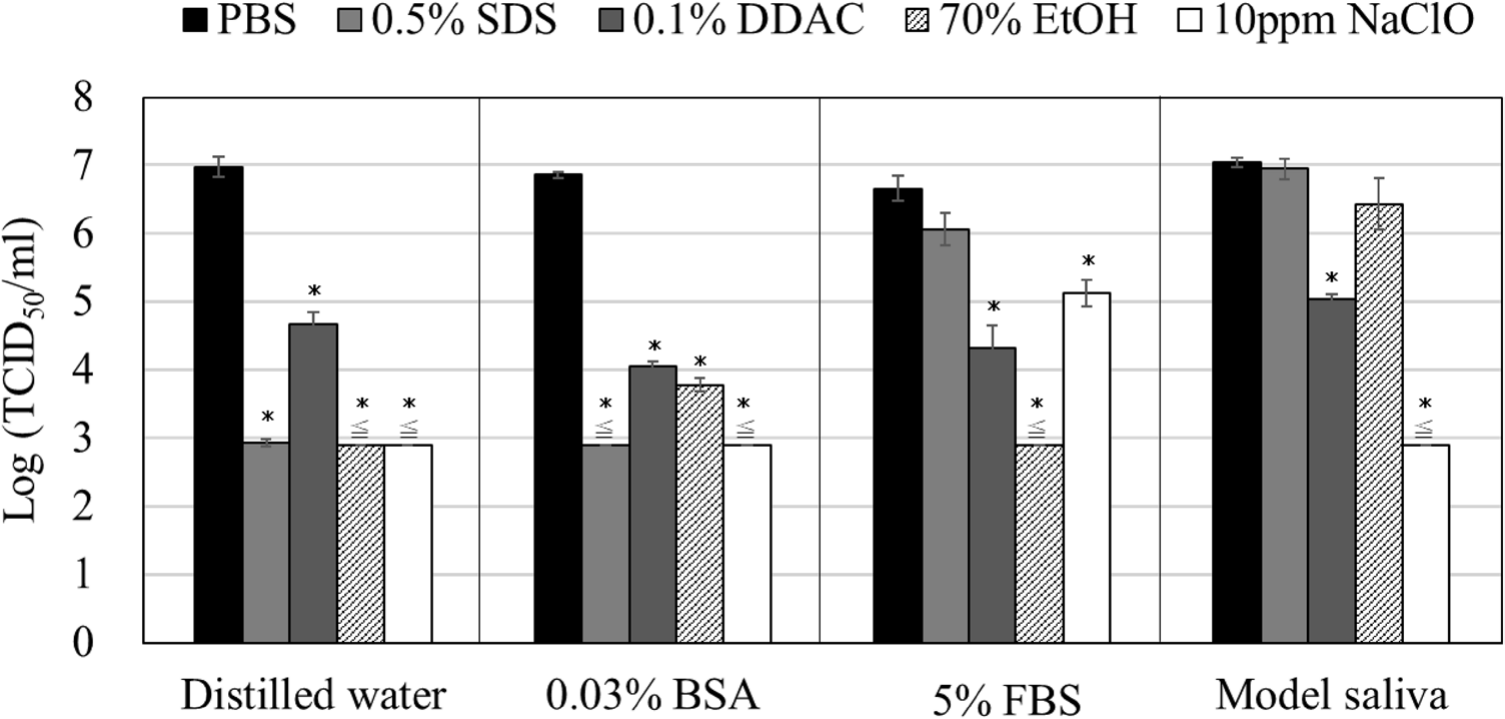
Additive effect of environmental contaminants on feline calicivirus (FCV) inactivation agents. The contact time was 10 min, and the infectivity titer (log (TCID_50_/mL) was expressed as the mean ± SD (*n* = 3). The concentration of each component in the FCV suspension was equal to that in the reaction solution. *﻿Indicates statistically significant differences (*p* ≤ 0.05).

We also evaluated the effect of the agents in the presence of environmental contaminants, such as load substances (BSA and fetal bovine serum [FBS]), which are recommended by ASTM and CEN, and environmental contamination, such as model saliva (Fig. 1; Table 6)^15^. Compared to the results with FCV in DW, the effect of 0.03% BSA did not change significantly. In 5% FBS, the effects of SDS and NaClO significantly reduced, whereas those of DDAC and ethanol remained almost unchanged. When the FCV suspension was replaced with model saliva, the effects of SDS and ethanol significantly reduced, whereas those of DDAC and NaClO remained unchanged.

**Table 6:** Composition of the model saliva.

Ingredients	mM
KCl	13.5
NaHCO_3_	5.0
KSCN	2.5
NH_4_Cl	6.0
(NH_2_)_2_CO	3.3
KH_2_PO_4_	6.0
CaCl_2_	4.0
MgCl_2_·6H_2_O	0.2
Mucin from bovine submaxillary glands	3000 ppm

## Discussion

This study investigated the effects of media components in viral suspensions and potential coexisting environmental contaminants on the efficacy of viral inactivation agents. Herein, FCV suspensions including EMEM were replaced with DW, each component group in EMEM, or model saliva. There were no significant differences in the infectivity titers of FCV across the replaced suspensions, indicating that the replacement of dispersing agents had no effect on the infectivity of FCV.

The results of this study also revealed that components of EMEM influenced the efficacy of various viral inactivators. SDS showed no inactivation effect on FCV in EMEM but exhibited strong efficacy at a concentration of 0.5% w/v when the FCV suspension was replaced with DW. One novel finding of this study is that the inactivation efficacy of SDS decreases in EMEM but can be enhanced by replacing the FCV suspension with DW. As shown in Table 3, the inorganic salts and BAA in EMEM reduced the inactivation effect of SDS. Cannon et al. reported that 2% SDS had no inactivating effect when the FCV suspension was replaced with phosphate-buffered saline, although the reaction time was different^16^; this is similar to our finding that the effect of SDS decreased in the presence of inorganic salts. In contrast, DDAC showed greater inactivation at lower concentrations in EMEM than in DW. The inorganic salts and BAA in EMEM enhanced the inactivation effect of DDAC (Table 3). Thus, the influence of dissolved components in virus suspension on the surfactants varies depending on the type of surfactant used.

Ethanol exhibited comparable inactivation effects at concentrations of 50% v/v and 70% v/v in EMEM, with no significant differences observed. This result was consistent with the trend reported by Park et al.^17^. Conversely, compared to FCV in DW, ethanol showed minimal inactivation effects at low concentrations (≤ 50% v/v) but demonstrated strong inactivation activity at a concentration of 70% v/v. In the absence of EMEM, ethanol exhibited a significant inactivation effect when the contact duration was 10 min. As shown in Table 3, the reduced inactivation effect of 70% v/v ethanol in EMEM can be attributed to the inorganic salts present in EMEM. Furthermore, when protein-rich solutions such as BSA or FBS were introduced, the strong inactivation effect of 70% v/v ethanol was maintained, indicating that ethanol is minimally influenced by the presence of proteins.

Compared to FCV with DW, the inactivation effect of NaClO at low concentrations was reduced when using FCV in EMEM. The impact of various dissolved components, such as inorganic salts, amino acids, and other environmental contaminants such as proteins, on the inactivation effect of NaClO, is consistent with reports suggesting that its efficacy decreases in FCV suspensions containing Dulbecco’s modified Eagle’s medium, which has a higher amino acid content than EMEM. Additionally, studies suggest that highly purified FCV can be inactivated with low concentrations of free chlorine^8,9^.

The inactivation mechanism of non-enveloped viruses by surfactants is hypothesized to involve protein denaturation caused by the adsorption of surfactants onto the viral capsid, a protein structure on the viral surface^18^. Therefore, the CMCs of SDS and DDAC in the presence and absence of EMEM were compared (Table 4). As shown in Table 1, the inactivation efficacy of both SDS and DDAC increased against FCV in DW at concentrations above their CMC, suggesting that concentrations exceeding the CMC are crucial for effective FCV inactivation. At low concentrations, surfactants exist as monomers; however, as the concentration increases, the monomers become saturated and form self-aggregates, known as micelles. It is well established that the protein denaturation effect of surfactants is enhanced when they form micelles above the CMC, rather than acting as monomers^19^. Therefore, the improved inactivation effect at or above the CMC is believed to result from the enhanced adsorption of the surfactant to the viral capsid. It was presumed that, in the presence of EMEM, the CMC of both surfactants decreased, promoting their adsorption to the viral capsid, thereby enhancing their inactivation effect at lower concentrations. The CMC of DDAC decreased to approximately 0.018% w/v in the presence of inorganic salts, equivalent to that observed in the presence of EMEM, allowing it to exceed its CMC at a concentration of 0.05% w/v. This suggests that the protein denaturation effect of DDAC was enhanced in the presence of inorganic salts. Previous studies have demonstrated that inorganic anions, such as sulfate ions, can enhance the protein denaturation and FCV inactivation effects of cationic surfactants^18^. Similar effects were likely induced by the salts present in EMEM. In contrast, SDS demonstrated a lower inactivation efficacy in the presence of EMEM, even at concentrations above its CMC.

Examining the pH changes in samples based on differences in dispersants revealed that when DW was added to an SDS sample, the pH remained unchanged. However, when EMEM was added, the pH increased from 6 to 7, attributed to EMEM’s buffering capacity. Therefore, the efficacy of 0.5% w/v SDS adjusted to different pH levels using NaOH or HCl was evaluated in FCV in DW. The results revealed a tendency for reduced inactivation efficacy at pH 6.5 and above (Supplementary Fig. S1.). In the presence of EMEM, the buffering actions of BAA and inorganic salts, such as Na_3_PO_4_ and NaHCO_3_, shift the pH to 7–8, which likely reduces the efficacy of SDS. Therefore, when using surfactants as virus-inactivating agents, it is crucial to apply concentrations above the CMC and consider the impact of pH changes on their efficacy.

The inactivation effect of ethanol is attributed to considered to arise from its ability to agglutinate viruses^20,21^. Ethanol, which has a high affinity for water, is known to denature and agglutinate proteins by dehydrating the area around them; this solvent effect is commonly used for protein crystallization^22^. The addition of salts may promote aggregation; therefore, the optimal ethanol concentration and salt type may vary depending on the target protein. The inactivation effect of ethanol against FCV in DW was low at a 50% v/v concentration. However, it significantly increased at a concentration of 70% v/v, suggesting that the enhanced solvent effect contributed to the increased inactivation efficacy observed. However, in EMEM, the inactivation effect of 70% v/v ethanol was reduced, likely because of the components of EMEM, such as inorganic salts. Inorganic ions are thought to reduce the electrostatic repulsion between capsid proteins, thereby stabilizing their structure and diminishing the denaturation effect of ethanol. Therefore, when using ethanol to inactivate non-enveloped viruses, it is crucial to ensure a contact time of approximately 10 min, utilize a high concentration where aggregation effects become significant, and consider the influence of inorganic salts and other contaminating components.

NaClO inactivates viruses through its oxidizing action, and its efficacy depends on the concentration of free chlorine. The Centers for Disease Control and Prevention (CDC) recommend using at least 1000 ppm of free chlorine for disinfecting surfaces^23^. In our study, free chlorine was consumed by all components, except inorganic salts and glucose (Table 5). The addition of inorganic salts and glucose showed high inactivation effects similar to those observed with DW (Table 3). Amino acids, particularly BAA, reduce the efficacy of NaClO, indicating that the inactivation effect of NaClO varies significantly depending on the type of amino acids present. Considering the potential protective effects of contaminating components on viruses, it is crucial to utilize high concentrations of NaClO or apply it to targets from which the contaminants have been removed to achieve adequate inactivation efficacy.

Although the specific effects of each agent evaluated in this study vary, the inactivation effect on FCV is generally assumed to occur through the denaturation of the viral capsid protein and alterations in the overall capsid structure. It has been reported that norovirus VLPs are denatured and destroyed by sodium hypochlorite, while capsids aggregate with ethanol; however, the protein itself is not destroyed, as observed through TEM microscopy and VLP quantification^20^. To gain a deeper understanding of the effects of surfactants and the variations in effectiveness based on the combination of ingredients and bases, future studies should include structural analysis using FCV or its capsid, along with physical property analysis of the evaluation samples.

Moreover, we evaluated the efficacy of the virus-inactivating agents under conditions simulating environmental contaminants, such as protein- and droplet-based exposure, using model saliva (Fig. 1.). Serum typically contains approximately 6–8% protein and trace amounts of inorganic salts and other biomolecules, whereas model saliva primarily comprises inorganic salts and mucin^24^. For FCV in DW, low protein concentrations of these environmental contaminants, such as 0.03% BSA, did not affect the efficacy of the tested agents. In contrast, at high protein concentrations, such as 5% FBS, the presence of inorganic salts and biomolecules likely diminished the efficacy of SDS and NaClO. This reduction may be attributed to pH alterations caused by the buffering effects of biomolecules and inorganic salts and the consumption of surfactants and chlorine by proteins (Table 5). Conversely, DDAC and ethanol were less affected by the proteins and other components when the contact time was 10 min. In the case of model saliva, the efficacy of SDS and 70% v/v ethanol significantly reduced, likely due to the presence of inorganic salts and mucin. DDAC, however, DDAC exhibited relatively consistent efficacy even in the presence of inorganic salts. NaClO is not consumed by inorganic salts and is predominantly used for mucin oxidation. However, as free chlorine acts on both FCV and mucin, NaClO is believed to retain its inactivation efficacy under these conditions.

Further analyses revealed that, in addition to proteins, BAA and inorganic salts significantly influence the efficacy of virus-inactivating agents. Considering that actual environmental conditions are likely to involve contaminants other than proteins, evaluating the addition of individual proteins, such as BSA, under environmental contaminations conditions, may be insufficient. Instead, systems incorporating various contaminants, such as sera containing diverse components, are considered to be more suitable for efficacy testing. Furthermore, the evaluation of coexisting model saliva systems designed to simulate droplet transmission demonstrated that while the addition of inorganic salt groups, such as DDAC, could enhance virus inactivation efficacy, the presence of mucin and inorganic salts, as observed in the model saliva, negated this effect. This observation underscores that the efficacy of inactivating agents can vary depending on the combination of dissolved components. Collectively, these findings indicate that the effectiveness of inactivating agents is highly dependent on the state of the viral suspension used, with variations arising from differences in the mechanisms of action of each agent. Therefore, to accurately evaluate the antiviral efficacy of such agents, it is crucial to consider their actual usage conditions and assess the influence of expected environmental contaminants.

The following limitations of this study should be considered. First, the present results are based on the viral inactivation effect at specific concentration with a 10-min contact time to ensure a consistent comparison of all agents, reflecting typical usage conditions of detergents containing surfactants. However, the effectiveness is expected to decrease with shorted contact times. The actual environmental contaminants are composed of more complex components than media such as EMEM, BSA, or model saliva. Therefore, we recommend that considering the following points when replicating the actual conditions for the use of inactivating agents:

1. Test method: Select an appropriate test method based on the evaluation objective, such as an antiviral test in suspension, an antiviral test on hard surfaces, a virus removal test on hands, or a virus removal test on textiles (adjust as necessary).
2. Sample concentration: Determine a concentration that accounts for the dilution of the sample during practical use.
3. Contact time with the virus: Set the contact time that aligns with the expected real-world usage conditions.
4. Potential contaminants: For kitchen applications, select food-derived contaminants (such as oil and food residues). For hard surfaces, select contaminants, such as sebum and dirt.

Second, when developing products based on these agents, the influence of additives incorporated into the products must be taken into account. The findings of this study are expected to serve as a reference to enhance the inactivation efficacy of such products. Finally, this report is focused on the effects of the agents on FCV. Confirmation is required to determine if comparable effects occur with other non-enveloped viruses. Therefore, we are currently conducting tests and will report the findings as soon as they are available.

## Conclusion

In conclusion, this study verified that the efficacy of virus inactivation agents was affected by the presence of EMEM in the FCV suspension, and by the dissolved components of EMEM and environmental contaminants. Even with a simple dispersant replacement method using a column, the effect of each dispersant was successfully evaluated using FCV in DW, enabling the identification of the inherent efficacy of each investigated agent. It is crucial to accurately evaluate the performance of a virus-inactivating agent in DW, particularly when considering its practical applications, such as cleaning norovirus-contaminated surfaces from toilet flushes. As the effect of the agents is highly dependent on the conditions of the virus suspension, developing an evaluation system that considers the actual use of the agents and the influence of environmental contaminants is important to accurately predict the efficacy of virus inactivation.

## Methods

### Cell culture

Crandell–Rees Feline Kidney (CRFK) cells (CCL-94) were purchased from the American Type Culture Collection (ATCC). The cells were cultured in Roswell Park Memorial Institute-1640 medium (1×) (Thermo Fisher Scientific Inc.), supplemented with 1% penicillin and streptomycin (PS; Sigma-Aldrich) and 10% fetal bovine serum (FBS; Sigma-Aldrich) at 37 ℃ in the presence of 5% CO_2_.

### FCV propagation

FCV F-9 (VR-782) was obtained from ATCC and propagated in CRFK cells. To prepare the virus, FCV was added to a subconfluent monolayer of CRFK cells, which was washed three times with PBS(-) (Fuji Film Wako Pure Chemical Co., Ltd.) and replaced with EMEM (Fuji Film Wako Pure Chemical Co., Ltd.) supplemented with 1% PS. The cells were infected with FCV at a multiplicity of infection of 0.1 and incubated at 37 ℃ under a 5% CO_2_ atmosphere for 1 d. After confirming cell degeneration due to FCV proliferation, the virus was released through three freeze-thaw cycles (from − 80 ℃ to 25 ℃). To eliminate cell-derived components such as the cytoskeleton and nuclei, the viral suspension was centrifuged at 3000 ×*g* for 15 min, followed by centrifugation at 4000 ×*g* for 30 min, and then at 10000 ×*g* for 30 min. The supernatant obtained was used as an FCV evaluation sample containing EMEM with an infectivity titer of 1.3 × 10^8^ TCID_50_/mL.

### Replacement of FCV suspensions with different dispersions

A PD-10 column (Global Life Sciences Technologies Japan K.K.) was used to remove EMEM-derived dissolved compounds from the FCV evaluation sample. This column allows the separation of high-molecular-weight (≥ 5000 g/mol) from low-molecular-weight substances (≤ 1000 g/mol) in EMEM and enables the replacement of the sample dispersion medium with other media. The PD-10 column was filled with different dispersion media, such as sterile distilled water (Otsuka Pharmaceutical Co., Ltd.), and 2.0 mL of FCV suspension containing EMEM was applied to the column. FCV was desalted and exchanged with the dispersion media and was most frequently detected in the 2.0 to 5.0 mL fraction (data not shown). A total of 3.0 mL of the suspension was used as the FCV suspension, and this replacement was performed immediately before use to prevent aggregation. The infectivity titer was 9.7 × 10^7^ TCID_50_/mL when EMEM was replaced with DW.

### Preparation of FCV suspensions including each dissolved component included EMEM

The influence of each dissolved component was examined separately for EMEM and its major components. The most abundant components in the EMEM formulation were divided into four groups: inorganic salts, BAA, NAA and others (Table 2)^24^. Aqueous solutions in each group were adjusted with ultrapure water (Milli-Q). The FCV suspension, including the inorganic salt group at the same concentration as EMEM, was prepared using a PD-10 column. The other FCV suspensions were prepared by mixing FCV in DW suspension containing each component to match the concentration of the EMEM formulation. The average infection titer was 9.8 × 10^7^–1.8 × 10^8^ TCID_50_/mL.

### Preparation of FCV suspensions including environmental contaminants

As environmental contaminants in this experiment, we used BSA (> 98%, Sigma-Aldrich), a protein recommended for use by CEN and ASTM, and the FBS used for CRFK culture as bovine serum^25,26^. Briefly, dispersions containing each contaminant were mixed with FCV in DW, adjusting the concentrations to 0.03 and 5% for BSA and FBS, respectively, in the evaluation sample. FCV suspensions, including the model saliva, were prepared by mixing with FCV in DW (Table 6). The average infection titer was 5.0 × 10^7^–1.1 × 10^8^ TCID_50_/mL.

### Inactivation agents of FCV

SDS (Nacalai Tesque, Inc.), DDAC (Lion Specialty Chemicals, Inc.), ethanol (Kanto Chemical Co., Ltd.), and NaClO (Fuji Film Wako Pure Chemical Industries Co., Ltd.) were purchased from the corresponding manufacturers. Each sample was adjusted using Milli-Q water and evaluated for the effect of concentrations used in actual personal care cleaners and other products; the concentration range of SDS was 0.05–0.5% w/v, that of DDAC was 0.01–0.1% w/v, that of ethanol was 30–70% v/v and that of NaClO was 10–100 ppm.

### Virus inactivation assays

The virus inactivation effects were evaluated using the 50% tissue culture infectious dose (TCID_50_) method. Under room temperature (20 ± 5°C), 0.05 mL of an FCV suspension was exposed to 0.45 mL of the sample solution of the inactivation agents for 10 min. Then, 0.1 mL of the mixture of the FCV and the inactivation agent solution was added to 0.9 mL of Soybean-Casein Digest Broth with Lecithin & Polysorbate 80 ‘’DAIGO’’ (SCDLPB, SHIOTANI M.S. Co.,Ltd) for neutralization. This solution was immediately serially diluted in the culture medium,　which was EMEM supplemented with 1% PS and inoculated into CRFK cells prepared in 96-well microplates cultured at 37 ℃ with 5% CO_2_. PBS(-) was used as the control solution. The logarithmic decrease in infection titer (Δlog) was calculated from the difference between the logarithmic values of the control and the evaluated samples.

### Determination of the CMC of the surfactants

The CMCs of the surfactants were determined by measuring their static surface tension using the Wilhelmy plate technique with a force tensiometer (K100C, KRUSS GmbH). Specifically, the CMCs were determined from the breaking point of the surface tension versus the logarithm of the concentration curve at 25 ± 0.5 ℃.

### Determination of the free Chlorine concentration from NaClO

Aqueous solutions containing various dissolved compounds at each test concentration without FCV were mixed with a NaClO solution at a ratio of 1:9 and incubated for 10 min. The free chlorine concentration in the mixed samples was measured using a Digital Pack Test for residual chlorine (Kyoritsu Chemical Laboratory, Inc.). The amount of chlorine consumed was calculated as the difference between the values of chlorine mixed with water and those mixed with each compound.

## Electronic supplementary material

Below is the link to the electronic supplementary material.

Supplementary Material 1

## Data Availability

Data is provided within the manuscript or supplementary information files.
