# Supplementary material for: Influence of cell culture-derived media and environmental contaminants on the effect of feline calicivirus inactivation agents
Source: Sci Rep. 2025 Jul 1;15:21365. doi: 10.1038/s41598-025-05311-7 (PMC12216461; doi:10.1038/s41598-025-05311-7)

**Figure Legends:**

**S1 Effect of pH on FCV inactivation effect of SDS**

The contact times was 10 minutes, and the inactivation effect was expressed as the mean ± SD of n = 3. The FCV suspension was replaced with DW. The pH of the SDS was adjusted using HCl or NaOH. ^*^*p* ≤ 0.05

**【S1】**


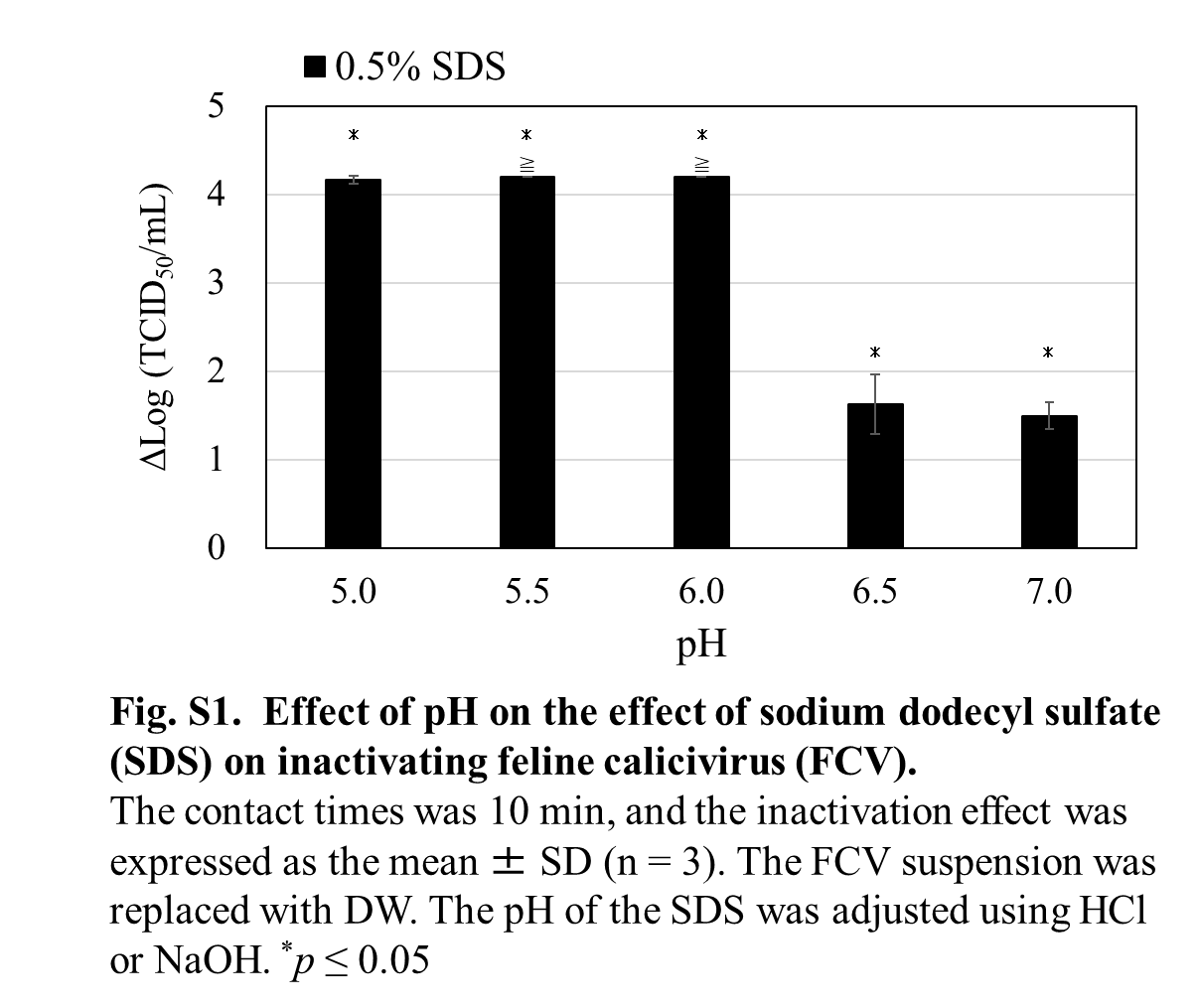

Supplement: Supplementary file 1 — Supplementary Material 1 [file 41598_2025_5311_MOESM1_ESM.docx]
